# Hedgehog signaling pathway is an influential factor on vascular biology: a review

**DOI:** 10.1007/s13105-025-01113-7

**Published:** 2025-08-02

**Authors:** Mi Ai, Li Xiao, Yilin Yu, Laidi Wu, Ollie Yiru Yu, Yingguang Cao, Jianmiao Liu, Ke Song

**Affiliations:** 1 Department of Stomatology, Tongji Hospital, Tongji Hospital Medical College, Huazhong University of Science and Technology, Wuhan, 430030 China; 2 Department of Prosthodontics and Implantology, School of Stomatology, Tongji Medical College, Huazhong University of Science and Technology, Wuhan, 430030 China; 3 Hubei Province Key Laboratory of Oral and Maxillofacial Development and Regeneration, Wuhan, 430022 China; 4 Faculty of Dentistry, The University of Hongkong, Hong Kong, SAR China; 5 Cellular Signaling Laboratory, Key Laboratory of Molecular Biophysics of Ministry of Education, Huazhong University of Science and Technology, Wuhan, China

**Keywords:** Hedgehog, Vasculogenesis, Angiogenesis, Arterial remodeling, Gli1^+^MSCs

## Abstract

Hedgehog (Hh) signaling is an important pathway involved in major biological processes such as embryonic development, adult morphogenesis, and vascular biology (i.e., vasculogenesis, angiogenesis and arterial remodeling). The latter role was more recently elucidated, occurring through regulation of angiogenic cytokines and controlling the proliferation, and migration of endothelial cells (ECs) or vascular smooth muscle cells (VSMCs), that help deliver oxygen and nutrients to tissues. Anomalous inhibition or activation of Hh signaling is therefore implicated in various pathological conditions, including vascular diseases. However, the mechanisms of Hh involvement in vascular biology have not been systematically clarified. This review covers recent research regarding the regulatory role and mechanism of Hh signaling in vasculogenesis, angiogenesis, and arterial remodeling. We conclude that the Hh signaling pathway holds great promise for treating vascular diseases and cancers. We encourage further research to develop a full understanding of the underlying mechanisms so that we can better determine the Hh pathway’s therapeutic value.

**Graphical abstract:**

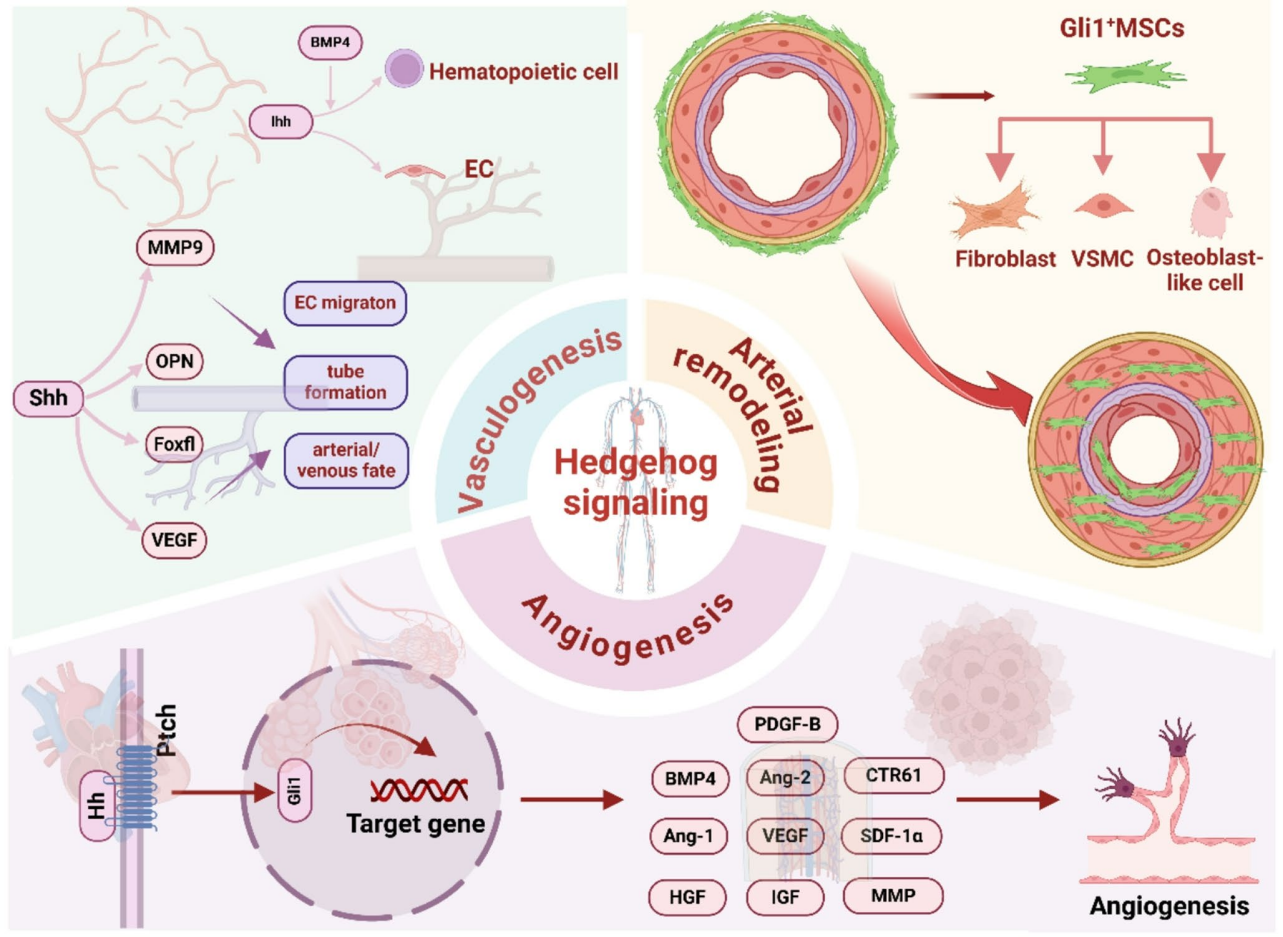

## Introduction

**Fig. 1:**
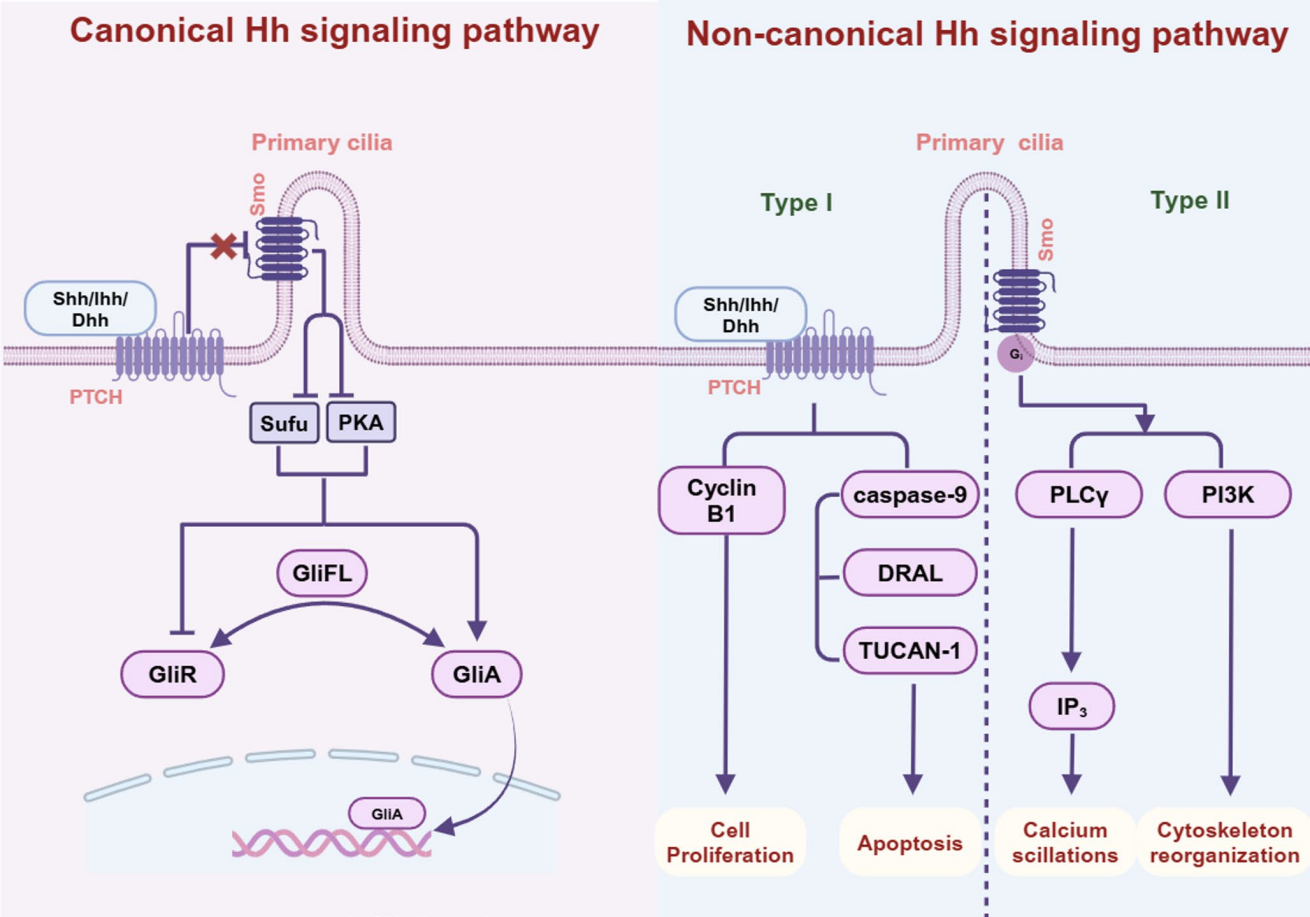
Canonical and the non-canonical Hh signaling pathways

First reported in 1980, Hedgehog(Hh) signaling plays a pivotal role in regulating organ development, tissue homeostasis, repair and regeneration [74]. The Hh signaling pathway comprises three HH ligands, Sonic hedgehog (Shh), Indian hedgehog (Ihh), Desert hedgehog (Dhh), a pair of 12-span transmembrane receptor proteins, patched (Ptch)1 and ptch2, a 7-span transmembrane protein: Smo and three transcription factors: Glioma-associated oncogene homolog (Gli)1, Gli2, Gli3 [40]. Moreover, it is well established that Hh signaling transduction occurs via the primary cilia on the cell surface. Classically, in the absence of a Hh ligand, Ptch1 suppresses the activity of Smoothened (Smo), and the binding of Hh ligands to Ptch releases Smo from the inhibition of Ptch in the primary cilia. Smo then activates Suppressor of Fused (Sufu) and Protein Kinase A (PKA), causing a conformational change in the truncated repressor form (GliR) to the transcriptional activator form (GliA). The latter then translocates into the nucleus and triggers Hh signaling (Fig. 1) (Dunaeva & Waltenberger [21, 42]; Xu, Iyyanar, Lan, & Jiang [106]). Subsequent research has found Gli-independent mechanisms can also activate Hh proteins. This “noncanonical Hh signaling, can be divided into: Type I (no Smo needed), and Type II (Smo functions without Gli transcription factors). Type I non-canonical Hh signaling also participates in apoptosis via recruiting caspase-9, DRAL, and TUCAN-1 [62], as well as in cell proliferation via regulating Cyclin B1 (Barnes, Kong, Ollendorff, & Donoghue [3]. Brennan, Chen, Cheng, Mahoney, & Riobo [5]). Type II non-canonical Hh signaling has different roles depending on whether the Gli/Smo complex activates PLCγ or PI3K, one leads to calcium oscillations and the other, cytoskeleton reorganization (Fig. 1) (Dilower et al., [17]).

**Fig. 2:**
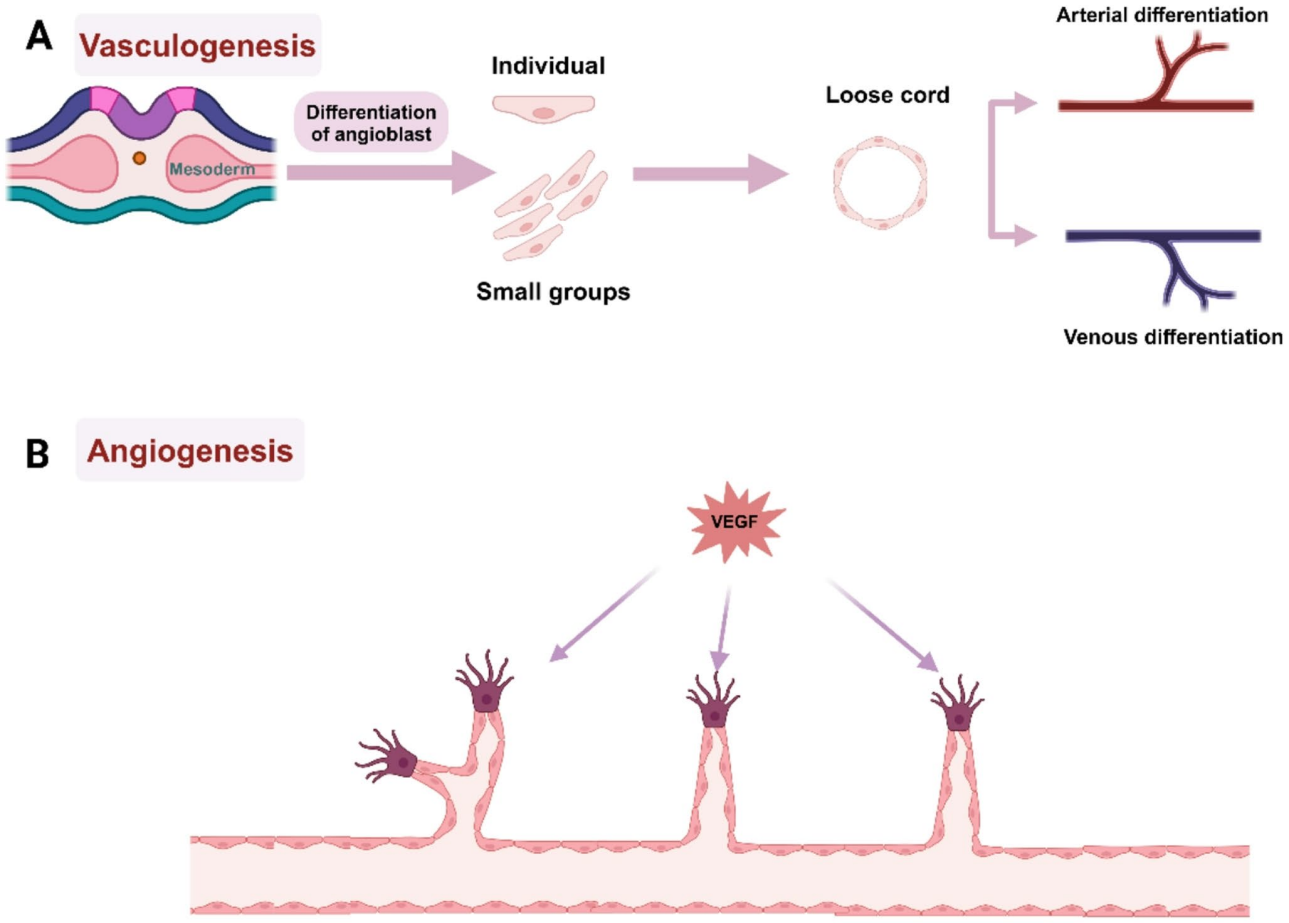
Schematic showing vasculogenesis, angiogenesis

The vascular system is important to embryonic survival, organ development, and maintaining organ function post-injury. The angioblast cells begin to differentiate and separate in the mesoderm and the separated cells migrate either individually or in groups to specific sites and adhere to one another to form loose cords in a unique way. These cords then mature into embryonic vessel, this process is called vasculogenesis (Fig. 2A) (Poole & Coffin [81]). Throughout life, new branches then grow from existing blood vessels through the process of angiogenesis [99]. Mechanistically, vascular endothelial growth factor (VEGF) action causes ECs migration to tip cells, where they form buds from existing vessels (Fig. 2B) (Negri, Faris, Berra-Romani, Guerra, & Moccia [71]).

**Fig. 3:**
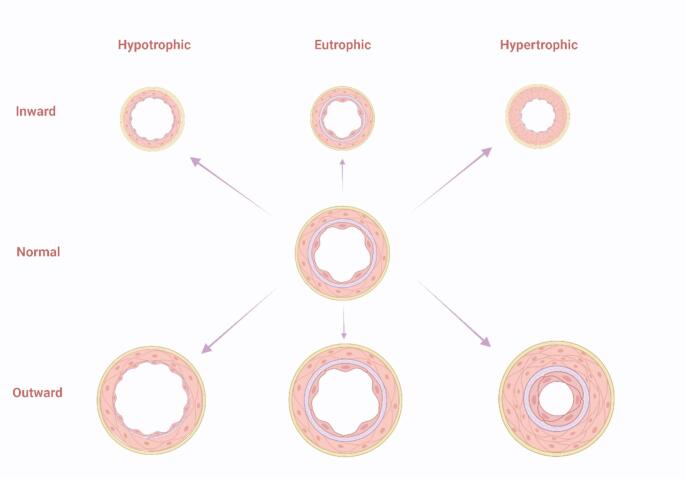
Schematic showing arterial remodeling

In response to biochemical and biomechanical stimuli, blood vessels then undergo structural and functional changes to the vascular wall in arterial remodeling [98]. Arterial remodeling is characterized by changes in the structure and function of the vascular wall, and it can be categorized into two types: atherosclerosis and arteriosclerosis [98]. Atherosclerosis is early characterized by leukocyte recruitment and pro-inflammatory cytokines expression [58], and arteriosclerosis is closely associated with the aging process and often linked to systemic cardiovascular, metabolic, or inflammatory disorders [98]. Moreover, arterial remodeling can manifest in two directions—either inward or outward—and can be characterized by hypertrophic changes (thickening of the vascular wall), eutrophic conditions (maintaining constant wall thickness), or hypotrophic alterations (thinning of the vascular wall) (Fig. 3) (Mulvany et al., [66]).

Hh signaling is essential for correct vascular system development and is thus also implicated in blood vessel formation under pathological conditions. Inhibition of Hh activity completely restrains vessel formation (Moran, Myers, Lewis, & Krieg [64]), but deletion of a negative Hh regulator called Patched1 (Ptch1) also reduces vessel density [14]. Thus, targeting Hh signaling has great potential as a therapeutic strategy for vascular diseases. Previously, we showed that that inhibiting Hh signaling in Gli1-positive mesenchymal stem cells (Gli1^+^MSCs) suppresses their differentiation into myofibroblasts, thereby alleviating vascular fibrosis [92]. Additionally, Shh treatment significantly increased blood vessel density in new bone regions to promote bone regeneration (Song, Rao, Chen, Huang, & Cao [93]).

In this review, we comprehensively summarized the pivotal role of Hh signaling in vasculogenesis, angiogenesis, and arterial remodeling under physiological and pathological conditions.

## Hh signaling pathway in vasculogenesis

Vasculogenesis initiates in the embryonic stage around Day 18 (Diaz Del Moral, Barrena, Munoz-Chapuli, & Carmona, [16]). The early stage of vasculogenesis begins with the formation of blood islands containing haemangioblasts, which can differentiate into VEGFR2^+^vascular precursor cells-angioblast. And this process leads to the formation of endothelial cells (ECs) that line the interior walls of blood vessels [20]. Thus, angioblast differentiation and formation of primitive blood vessels (collectively, vasculogenesis) are major steps in vascularization [88]. During embryonic development, Ihh is an endogenous signaling molecule that plays a pivotal role in the development of the earliest stages of the hematopoietic and vascular systems. The Hh signaling pathway in the epiblast is activated by endodermal Ihh, subsequently increasing Ptch, Smo and Gli1 expression, which is solely sufficient to induce the formation of hematopoietic and endothelial cells under the regulation of Bone morphogenetic protein 4 (Bmp4) (Dyer, Farrington, Mohn, Munday, & Baron [23]). However, while Ihh plays a vital role in early hematovascular development, the role of Shh has received the most attention because it is considerably more potent than other proteins at activating Gli transcription factors (Shh > > Ihh > Dhh) [77], Shh is produced by choroid plexus epithelium of hindbrain, and its downregulation alters the shape of ECs from a “cobblestone” pattern to a swirling bundle of elongated cells (Chinchilla, Xiao, Kazanietz, & Riobo [11]). Additionally, Shh treatment significantly increases ECs migration rate, and promotes capillary morphogenesis through upregulating matrix metalloproteinase 9 (MMP9) and osteopontin (OPN) in ECs. The lack of involvement from Gli implies mediation by the non-classical Hh pathway rather than classical Hh pathways [85].

Shh is required for proper vessel formation and remodeling during embryonic blood-vessel development. When Shh signaling is attenuated by 5E1 (a Shh blocking antibody), the first and second aortic arches exhibit delayed remodeling, while defects of the anterior cardinal vein and its branches begin to exhibit defects that manifest as hemorrhages and endothelium-lined protrusions. The end result is a protruding anterior cardinal vein and outflow tract, delayed fusion of the dorsal aorta, impaired branching of the internal carotid artery, and delayed remodeling of the aortic arch (Kolesova, Roelink, & Grim [44]). Because angioblasts both expressed and responded to components of the Hh signaling pathway, any disruption or inhibition negatively affected tube size and continuity [99]. Furthermore, Hh morphogens trigger angioblast differentiation depending on concentration, with higher concentrations repressing venous cell fate, and tilting the trajectory toward arterial development. Hh protein is thus a critical factor in determining the arterial-venous contribution of angioblasts (Kohli, Schumacher, Desai, Rehn, & Sumanas [43]; Williams et al., [103]).

Bone morphogenetic proteins (BMPs) and VEGF are important to vascular tube formation. Endoderm-derived Hedgehog ligands (Ihh and Shh) induce Foxf1 expression in the mesoderm, which subsequently activates Bmp4 transcription; the resulting Bmp signaling then promotes vascular tube assembly from mesodermal progenitors [1, 4]. Additionally, Shh stimulation enhances VEGF expression in cultured fibroblasts to promote endothelial tube formation, Hh and VEGF are upstream of the Notch pathway in mouse embryos, which participates in the determination of arterial/venous fate [14, 80]. Hh also interacts with other pathways to mediate vasculogenesis or arterial vein specification, including the ERK/MAPK pathway, and PI_3_K/Akt pathways (Hong, Kume, & Peterson [36]). Despite the relevance of these interactions to vascular diseases, relevant studies are lacking (see a summary in Table 1). Greater attention to these Hh-linked vasculogenesis pathways would benefit the development of targeted therapeutic strategies.

**Table 1:** Role of Hedgehog signaling in vasculogenesis

Intervention	Cell type/Animal model	Results	Conclusion	Reference
Blocking Ihh function	CD1 or ICR mice	Inhibit vasculogenesis	Ihh from primitive endoderm activates Hh signaling in epiblasts is crucial for vasculogenesis	[23]
Application of Shh	Human umbilical vein (HUVEC), human cardiac microvascular endothelial cells (HMVEC), bovine aortic endothelial cells (BAECs)	1. Shh promotes change to endothelial cell shape from “cobblestones” to elongated swirls oriented in bundles 2. Shh significantly increases endothelial cell migration rate, and promotes capillary morphogenesis 3. Upregulated MM9 and OPN expression	Hh proteins promotes vasculogenesis via regulating EC migration and proliferation through non-canonical pathways	[11, 77, 85]
Inhibition or activation of Shh and Smo	C57BL/6 mice, Fertilized quail egg, Zebrafish	1. Protrusion of anterior cardinal vein and outflow tract, delayed fusion of dorsal aorta, impaired branching of internal carotid artery, and delayed remodeling of aortic arch 2. Arterial differentiation is more likely with higher concentration	Hh signaling is crucial for vascular development, and angioblast trajectory (to artery or vein)	[43, 44, 103]
Hedgehog kockout	Smo^−/−^ mice, Human embryonic stem cells (hESC), Ptch1^flox/flox^ mice, Zebrafish	1. To form primitive vascular networks, Hedgehog proteins activate forkhead transcription factor (Foxf1) to promote Bmp4 expression in mesoderm 2. Hh is required for VEGF expression. Both Hh and VEGF activate Notch pathway, necessary for arterial/veno-us identity 3. Hh signaling interacts with other pathways (e.g., ERK/MAPK, PI_3_K/Akt) to mediate vasculogenes-is or artery-vein specification	Hh signaling does not regulate vasculogenesis independently	([1, 4, 14, 36, 60])

## Hh signaling pathway in angiogenesis

### Under physiological conditions

Hh signaling orchestrates angiogenesis during organ development. Of the morphogenic proteins included in the Hh family, Shh involved in lung development is the most studied. Shh-deficient mice exhibit abnormal pulmonary vasculature that results in lung-branching defects [97]. For example, lung specific deletion of Shh during embryonic day (E) 11.5–13.5, hampered vascular bed development in response to fewer VSMCs and ECs. Shh deletion on E12·5 hampered vascular and airway branching for 4–6 days [63]. During this period, total angiopoietin-1 (Ang1) expression in lung was downregulated, indicating a defect in the Ang/Tie2 signaling pathway, and this impaired the vascular stabilization [97]. Shh coordinates with fibroblast growth factor 9 (FGF9) to regulate VEGFA indirectly, an essential factor in pulmonary vessel development, however Shh and FGF9 signaling are transmitted to the lung mesenchyme but not to endothelial cells (White, Lavine, & Ornitz [102]). Thus remarkable advancements have shed light on this problem, paracrine Shh signaling from pulmonary epithelium programs mesenchymal Foxf1 expression via Gli-mediated transcriptional activation, establishing the molecular framework for developmental angiogenesis (Chapouly, Guimbal, Hollier, & Renault [8]. Seo, Kim, Park, Kim, & Cho [90]).

In addition to pulmonary vascular development, Hh signaling is also an essential regulator of coronary vascular development. Specifically, the Hh signaling pathway is activated by potent angiogenic inducers called FGFs. Upon activation, Hh signaling then upregulates VEGFA, VEGFB, VEGFC, and angiopoietin-2 (Ang-2) expression in cardiomyoblasts which generates a paracrine signaling cascade targeting coronary endothelial cells [67]. This process is essential for normal coronary vessel development [52, 53].

Blood vessels affect cartilage conversion to bone via inducing chondrocyte proliferation, maturation, and hypertrophy (Yin, Gentili, Koyama, Zasloff, & Pacifici [109]). It has been realized that VEGFA orchestrates perichondrial vascularization and osteoblast differentiation through increasing the expression of Ihh and inhibiting Notch2 in perichondrial cells, which plays a pivotal role in bone development [19]. Ihh is a promising regulator of bone development, including chondrocyte maturation and cartilage vascularization. Analysis of Ihh^−/−^ mice revealed an absence of blood vessels that eventually led to cartilage matrix degradation ([13]; Hilton, Tu, Cook, Hu, & Long [35]). Ihh inhibitor Gli3 participates in chondrocyte differentiation via regulating the level of parathyroid hormone-related protein (PTHrP) (Hilton et al., [35]). When Gli3 was removed from Ihh^−/−^ mice, however, cartilage vascularization was not completely rescued (Koziel, Wuelling, Schneider, & Vortkamp [45]). Later research then allowed us to conclude that Gli2 could induce cartilage vascularization even after Gli3 removal, therefore, Gli2 was sufficient for Ihh signaling during cartilage development [41]. Another notable function of Shh is the regulation of angiogenesis during bone formation. In co-cultures of primary osteoblasts and outgrowth endothelial cells, Shh promotes the formation of microvessel-like structures via upregulating Ang-1, Ang-2, and VEGF [18]. This process is essential for modulating perichondrial vascularity and osteoblast differentiation [19, 27].

Furthermore, Hh signaling stimulates craniofacial bone development via regulating microvascular morphogenesis. Inhibiting Hh signaling during embryonic development, causes distortions to craniofacial vasculature. These changes are likely associated with the downregulation of VEGF and bone morphogenetic protein 4 (BMP4) (Nagase, Nagase, Yoshimura, Machida, & Yamagishi [70]). Concomitantly, the Shh pathway in cranial neural crest cells (cNCCs) stabilizes the endothelial cord network. Inhibiting Shh disrupts microvascular morphogenesis, leading to orofacial clefts and holoprosencephaly (Nagase, Nagase, Osumi, et al., [68, 70, 95]). Likewise, the specific MSC marker Gli1 is a vital transcriptional factor; tracing Gli1^+^MSCs could therefore clarify Hh involvement in bone formation. Improvements in experimental techniques have led to the discovery that Gli1^+^MSCs are spatially associated with osteogenesis and angiogenesis, a link that warrants further study [59, 87].

The Hh signaling pathway is also involved in blood vessel development of other organs. Notably, neural tube angiogenesis is dependent on Shh signaling through Ang-1 expression (Nagase, Nagase, Yoshimura, Fujita, & Koshima [69]). When choroid plexus is functioning normally, Shh is pivotal for vascular outgrowth [72]. Specifically, Shh-Gli1 signaling is necessary for skin expansion via the pathway’s involvement in angiogenesis, epidermis thickening, and thinning of dilated skin [111]. Targeting the Hh signaling pathway thus appears to be a potential strategy for regulating organ vasculature development. Table 2 summaries available studies on the role of Hh signaling in regulating angiogenesis under physiological conditions.

**Table 2:** Hedgehog signaling in angiogenesis under physiological conditions

Anatomical position	Intervention	Cell type/Animal model	Conclusion	Reference
Lung	Shh or Smo deletion	Shh^−/−^mice, Shh/Tie2-LacZ mice, Smo^fl/fl^ mice	Shh deletion at different developmental stages results in air branching defects	[63, 90, 97, 102]
Coronary vasculature	Shh activation or inhibition	Gli2/αMHC-ER-Cre mice	FGFs activated Hh signaling pathway and then increasing the expressing of VEGFA, VEGFB, VEGFC, angiopoietin-2 (Ang-2) to regulate coronary vasculature development	[51, 53, 67]
Endochondral bone	Ihh, Gli3, Shh deletion	Ihh^+/−^;PtchLacZ mice, Vegfa^fl/fl^;Osx-Cre GFP mice, C57BL/6 mice, Gli3^Xt − J^ mice, Chondrocyte,	1. One way in which Ihh involved in endochondral bone development is through mediating vessel expand into cartilage matrix 2. Ihh effectors Gli2 and Gli3 respond to vasculature-derived signals and influence osteogenesis 3. Shh promotes formation of microvessel-like structures through upregulating Ang-1, Ang-2, and VEGF	[13, 18, 19, 27, 35, 41, 45, 109]
Craniofacial bone	Shh inhibition	Gli1-CreERT2;Ai14 mice, Cdh5-Cre mice, ICR mice, C57BL/6	1. Hh signaling involves microvascular morphogenesis through up-regulating VEGF and BMP4 2. Shh pathway in cranial neural crest cells(cNCCs), stabilizes the endothelial cord network Gli1 + MSCs are spatially associated with osteogenesis and angiogenesis	[59, 68, 70, 87, 95]
Neural tube	Block Shh signaling	ICR mice	Neural tube angiogenesis is dependent on Shh signaling, which regulates Ang-1 expression.	[69]
Hindbrain choroid plexus	Shh knockout	Shh^flox/flox^ mice	Shh (from hindbrain choroid plexus epithelial cells) needed for vascular outgrowth	[72]
Skin	Inhibit Gli1	Mesenchymal cell	Shh-Gli1 signaling is involved in angiogenesis thickening epidermis and thinning the dilated skin	[111]

### Under pathological conditions

#### Hh signaling in ischemia diseases

Hh signaling has been strongly implicated in diseases affecting ischemic tissues, including the heart, muscle, brain, and hind limbs.

Acute or chronic blockage in coronary arteries causes heart ischemia [76]. Neoangiogenesis of the plaque causes it to rupture and move; the lack of stability is the primary reason that blockages are difficult to address. Experiments have revealed that this neoangiogenesis occurs under an anomalous response of the Hh pathway to ligands, moreover, insulin resistance adipocyte-derived exosomes (IRADEs) promote plaque burden and plaque vulnerability partly by inducing vasa vasorum angiogenesis, which is through Shh [82, 100].Thus, downregulating Hh can stabilize plaques. However, Hh signaling must be activated to relieve cardiac ischemia via appropriate angiogenesis to support the survival of small coronary arteries and capillaries (Lavine, Kovacs, & Ornitz [50]). Mechanistically, Hh signaling (specifically by Shh) appears to be activated by ischemia-indeuced hypoxia [39]. Shh does not directly regulate angiogenesis by acting on endothelial cells, but rather indirectly upregulates the expression of angiogenic growth factors in interstitial mesenchymal cells, such as platelet derived growth factor (PDGF)-B, VEGF-A, hepatocyte growth factor (HGF), and insulin-like growth factor (IGF) [80]. Moreover, Shh can also promote the involvement of VSMCs autophagy in cardiac repair [30, 56]. Corroborating that idea, after Shh gene transfer, fibrosis and cardiac apoptosis noticeably decreased after Shh gene transfer [49]. These studies strongly suggest that the Hh signaling pathway could serve as a promising therapeutic target for ischemic heart disease.

Importantly, restoring blood supply is not necessarily conducive to recovery; myocardial ischemia-reperfusion can lead to heart failure and even multiple organ dysfunction syndrome [105]. Angiogenesis plays a crucial role in myocardial ischemic-reperfusion, therefore, Shh, coordinates angiogenesis by promoting cardiac microvascular endothelial cell proliferation and decreasing its apoptosis, concomitant with the increasing in VEGF and FGF [29]. Shh merits further investigation for its ability to protect cardiomyocytes [78].

Ischemic stroke has a high mortality rate. Several studies have proposed that Shh has considerable therapeutic potential for strokes, in part by promoting angiogenesis. In cerebral ischemia, Shh promotes EC proliferation, migration and tube formation via increasing VEGF expression. The process appears to occur through Shh stimulating the RhoA/ROCK pathway to mediate angiogenesis [33]. Elevated angiogenesis led to an improvement in neurological scores and behavioral outcomes [10, 38].

Hh signaling can regulate muscle regeneration through angiogenesis in hindlimb muscle ischemia [79]. In particular, Shh increases capillary density and arteriole density to augment blood-flow recovery and limb salvage [80], concomitant with the upregulation of proangiogenic factors in ischemic muscle, such as including VEGF, Ang-1, and stromal-derived-factor-1α (SDF-1α) (Palladino et al., [75]). Furthermore, before muscle ischemia, the number of microparticles bearing Shh increased significantly; this microparticles are closely associated with angiogenesis in the ischemic limb [28]. These findings suggest that Shh is a promising target for treating ischemic injury. However, the absence of Shh actually led to a transient increase in angiogenesis of myocytes, indicating that Shh was not necessary for regulating ischemia-induced angiogenesis (Caradu, Guy, et al., [7]).

In contrast, Hh members Dhh and Gli3, have become a focus of research. Kruppel-like factor 2 (Klf2) plays a pivotal role in establishing and preserving the integrity of the endothelium. As downstream of Klf2, Dhh protects vascular integrity by maintaining EC function [6]. However, Dhh expression was downregulated in old mice both healthy and ischemic conditions, impairing muscle recovery, these conditions can be ameliorated through Dhh gene therapy, suggesting that Dhh has value as a therapeutic approach for ischemic diseases in the elder population (Renault, Robbesyn, et al., [83]). Gli3 also contributes to muscle repair during limb ischemia. Severely impaired ischemia-induced angiogenesis completely lacked Gli3, a coordinator involved in regulating EC migration and proangiogenic factors, including thymidine phosphorylase (TYMP) and Ang-1. AS a result, capillary density was low and repair was delayed [84]; Renault, Vandierdonck, et al., [86].

Collectively, Hh signaling is involved in recovery form most ischemic diseases. Extensive research on the pro-angiogenic effects of Hh signaling provides us with valuable data on this pathway as a promising therapeutic target for ischemic diseases.

#### Hh signaling in angiogenesis after injury

Generating new bone and maintaining constant blood supply are critical for post-fracture recovery [91]. The importance of Hh in angiogenesis can be seen in the upregulation of Gli1, and Ptch1 after stress fractures. Inhibiting Hh limits fracture callus blood-vessel density by 55% (Fuchs, Dohle, & Kirkpatrick [25]). In contrast, treatment with an agonist led to an 85% increase in callus blood-vessel density [61]. Moreover, healing time changed under inhibitor and agonist regardless of bone type (e.g., femur or skull) (Lee et al., [54]). In vitro experiments with engineered blood vessels revealed that Shh enhances bone formation and maturation [89]. Experimental studies then revealed that Gli1^+^MSCs are preferentially associated with CD31^hi^EMCN^hi^ vessels. During fractures, these cells initiate type H vessel formation through activating Gli and HIF-1α, thus mediating bone repair [9]. In summary, the data suggest that Hh signaling, is a pleiotropic pathway, that mediates bone formation and angiogenesis.

Hh signaling and the associated angiogenesis has been detected in other injured organs, including the cornea, nerves, and choroid. In both central and peripheral nerves, Hh related angiogenesis promotes neurological function and stabilizes the neurovascular unit [104]. Upon injury, Hh signaling induces Gli1^+^cells to distribute around blood vessels and accumulate at the injury site, promoting angiogenesis concomitantly with endothelial cells [24, 107]. Similarly, in post-injury cornea (Fujita, Miyamoto, & Saika [26]), choroid (H. He, Zhang, Li, Li, & Wang [32]. Nochioka et al., [73]), muscle [94], and skin [101]) activating Hh signaling controls angiogenesis and accelerates recovery. The important role of Hh pathways in tissue regeneration implies a strong potential for therapeutic applications.

#### Hh signaling in tumor angiogenesis

Dysregulation of the Hh pathway has been implicated in cancers, because of its ability to promote cell proliferation and regulate growth factor levels. For example, in oral squamous cell carcinoma (OSCC), Shh, Gli1, Gli2 and ptch1 are aberrantly expressed, but the antagonist cyclopamine reverses this phenomenon [48]. Evidence also exists to show that Hh pathway members besides Shh participate in tumor angiogenesis. In particular, Ihh and Dhh activity increased and modulated tumor growth ([2]; Y. Li, Liu, Wang, Wang, & Guo [57]).

Mechanistically, hyperactivation of Hh signaling increases proangiogenic factors, including VEGF, matrix metalloproteinase 2 (MMP2) and matrix metalloproteinase 9 (MMP9). Therefore, Hh signaling closely related to microvessel density [15]. In particular, the pro-angiogenic factor cysteine-rich angiogenic inducer 61 (CYR61), is an Hh signaling molecule that regulates angiogenesis and enhances malignancy in breast cancer (Harris, Pannell, Singh, Samant, & Shevde [31]). In addition to the hyperactivation of Hh signaling, tumor cells also exhibit ECs and infiltrating macrophages with elevated Shh, Ihh, and Gli1 expression, suggesting an involvement in angiogenesis and tumor metastasis [96]. Moreover, Shh secreted from pancreatic cancer cells enhances angiogenesis, specifically acting on endothelial progenitor cells and stimulating tube formation [108]. Therefore, triggering Hh signaling in tumor has potential as a cancer treatment. Table 3 summarizes studies on the role of Hh signaling in regulating angiogenesis under pathological conditions.

**Table 3:** Role of Hedgehog signaling in angiogenesis under pathological conditions

Disease model	Intervention	Cell type/Animal model	Conclusion	Reference
Cardiac ischemia	Shh gene transfer, Activation or of Hh signaling, Knockout Smo	Cardiac microvascular endothelial cells, H9c2 cell, Vascular smooth muscle cell, C57BL/6 mice, Smo^fl/fl^ mice, NLS-Ptch-LacZ mice, Wistar Kyoto rat	1. Ischemia-activated Shh facilitated angiogenesis through upregulating angiogenic growth factors (PDGF-B, VEGF-A, HGF, IGF) and promoting autophagy of vascular smooth muscle cells 2. Shh gene transfer increases angiogenesis to assuage left ventricular dysfunction; fibrosis and apoptosis decrease 3. Shh coordinates angiogenesis through accelerating cardiac microvascular endothelial cell proliferation and decreasing apoptosis in myocardial ischemic-reperfusion	[29, 30, 39, 49; 50, 56, 78, 80, 105]
Ischemic stroke	Activation or inhibition of Shh	Brain microvascular endothelial cells,	1. Astrocytes increase secretion of Shh promoting endothelial cell proliferation, and migration	[33]
Hindlimb muscle ischemia	Hh protein floxed	Shh^fl/fl^ mice, Smo^fl/fl^ mice, C57BL/6 mice, Gli3^+/−^mice C2C12 cell, Raw264.6 cell, human dermal microvascular ECs	1. Shh increases capillary density and arteriole density via upregulating VEGF, Ang-1, SDF-1α 2. Number of microparticles bearing Shh increases remarkably during PAD period 3. Shh is not necessary for regulating ischemia-induced angiogenesis 4. Dhh gene therapy ameliorates vessel and nerve density 5. Gli3 contribute angiogenesis through regulating endothelial cell migration and proangiogenic factors, including thymidine phosphorylase (TYMP) and Ang-1	[6; 7, 28, 75, 80; 83; 84, 86]
Fracture	Inhibition or activation of Hh signaling	Gli1-LacZ mice	1. In vitro, Shh enhances bone formation and maturation through developing engineered blood vessels 2. Gli1 + MSCs are preferentially associated with CD31^hi^EMCN^hi^ vessels, they initiate type H vessel formation	[9, 89]
other injury organ (cornea, nerve, choroid skin)		Gli1-CreERt2;tdTomato mice, C57BL/6 mice, Gli1CreER; R26RYFP mice, Brown-Norway rat, Sprague-Dawley rats, human umbilical vein endothelial cells, C2C12 cell, endothelial progenitor cells	Gli1 + cells distribute around blood vessels and accumulate at the injury site to promote angiogenesis, concomitant with endothelial cells	[24, 26, 32, 73, 94, 101, 104, 107]
Tumor	Inhibition or activation of Hh signaling	Human glioma cell line, MDA-MB-231cell, COS7 cell, endothelial progenitor cell	1. Shh, Gli1, Gli2, and ptch1 were each aberrantly expressed in tongue OSCC and in tumor microvascular cells 2. Hyperactivation of Hh signaling could increase VEGF, MMP2 and MMP9, CYR61 levels 3. Shh, Ihh, and Gli1 expression in endothelial cells and infiltrating macrophage increase 4. Cancer-cell-derived Shh enhances angiogenesis through specifically acting on endothelial progenitor cells and stimulating tube formation	[15, 31, 48, 96, 108]

## Hh signaling in arterial remodeling

Arterial remodeling is a reflection of the adaptation of vessel wall to biochemical and biomechanical stimuli, such as injury, hypoxia, diseases or aging. The development process of arterial remodeling include: the proliferation and differentiation of VSMCs, the degradation and fracture of elastin fiber, calcification and deposition of extracellular matrix material [98].

In addition to participate in vasculogenesis and angiogenesis, Hh signaling also play a pivotal role in arterial remodeling. Enhanced Shh signaling was observed in vascular smooth muscle cells (VSMCs) within the neointima of vein grafts harvested from mice experiencing restenosis [55]. The increasing expression of Shh can promote VSMCs growth and survival through VEGFA activation coordinating with Notch signaling [65]. Absolutely, arterial adventitial cells participate in neointima formation, it has confirmed that Shh and PDGF-BB can strongly increase the proliferation and migration of adventitial fibroblasts, which secrete interleukin-6 and − 8 to regulate the survival of VSMCs [22]. However, Shh was decreased in VSMCs during arterial calcification. Huang et al. have proposed that Shh inhibits the osteoblastic differentiation of VSMCs and can be reversed by overexpression of Gli2, which interacts with Runx2 to promote its ubiquitin proteasomal degradation [37].

Atherosclerosis accelerates arterial remodeling, with VSMCs migration and proliferation being critical to plaque development [98]. Considerable evidence supports the positive effect of Hh signaling on VSMCs survival. Experimental studies have confirmed that Shh promotes VSMCs autophagy and proliferation through activating AKT and G1 cyclin-retinoblastoma axis [55, 56]. Shh also participates in the transition from a contractile to a synthetic phenotype, leading to inflammatory release and vascular stenosis (X. Guo et al., [29, 110]).

Gli1 is not only a transcriptional activator of Hh signaling that regulates several biological processes, but also a vital marker for mesenchymal stem cells (MSCs). Thus, Perivascular Gli1^+^cells have the capacity to differentiate [47]. Indeed, Gli1^+^ cell differentiation is a key part of vascular remodeling. Typically, after acute injury, Gli1^+^ cells migrate from the adventitium to the media, and neointima, then differentiate into VSMCs. However, in the case of chronic kidney disease (CKD), Gli1^+^ cells differentiate into osteoblast-like cells to enhance vascular calcification [46]. Gli1^+^ cells can also develop into myofibroblasts that promote neointima formation during CKD through activating the PDGFRα and TGFβ1/SMAD pathways [92]. In addition to their differentiation ability, Gli1^+^ cells stimulate VSMCs induce hypoxia-induced pulmonary hypertension [34]. Gli1^+^ cells recruit and contribute to vascular muscularization in pulmonary hypertension induced by hypoxia, thus selective ablation of Gli1^+^ cells significantly alleviate vascular remodeling [12].

Overall, the application of Hh signaling in arterial remodeling diseases is a promising topic that warrants further investigation. Hh signaling-based treatments may be useful for maintaining normal vascular structures and functions. Table 4 summarizes studies investigating the role of Hh signaling in arterial remodeling.

**Table 4:** Role of Hedgehog signaling in arterial remodeling

Intervention	Cell type/Animal model	Conclusion	Reference
Shh gene transfer, Activation or of Hh signaling, Knockout Smo	Primary human vascular smooth muscle cell, mouse aortic smooth muscle cell, Sprague–Dawley rat, wistar rat, Gli1-CreERt2; R26tdTomato mice, C57BL/6 mice, Gli1CreERt2/PDGFRA^fl/fl^ mice	4. Gli1^+^cells participate in arterial remodeling via migrating from adventitial to media, neointima and differentiating into VSMC during acute injury 5. Gli1^+^cells differentiate into osteoblast-like cells to enhance vascular calcification in CKD 6. Gli1^+^cells differentiate into myofibroblasts promoting neointima formation through activating PDGFRα and TGFβ1/SMAD signaling. 7. Shh promotes VSMCs autophagy and proliferation through activation of AKT and G1 cyclin-retinoblastoma axis 8. Shh participates in the transition from contractile to synthetic phenotypes	[29, 46, 55, 56, 92, 110]

## Outstanding questions

Data in the literature has been suggested that Hh signaling participated in vascular biology, but the exact cellular and molecular mechanism of this process remains unclear: (1) it is a prerequisite to fully understand the precise origin of Hh pathway ligand proteins; (2) although it is known to be involved in regulating angiogenesis along with other pathways, but how exactly this network of pathways works still needs to be explored.

In addition, an increasing number of studies have implied MSCs play a vital role in arterial remodeling [34]; Kramann et al., [46]. Gli1^+^ cell as a kind of typical MSCs that regulates arterial remodeling through differentiating to VSMCs or osteoblasts-like cells. However, the exact origin of Gli1^+^cell remains unclear, only by identifying its source can therapies be better targeted.

Finally, it has been confirmed that the number of microparticles bearing Shh increased significantly before muscle ischemia, thus, detection of this microparticles may emerged as a reliable target for the diagnosis of ischemic diseases [28]. Moreover, inhibition or activation of Hh can regulated angiogenesis under pathological conditions, such as ischemia [79], or tumor. Thus, Hh signaling is a promising target to treat vascular-related diseases, but how to prolong its effective time and specific side effects should be the focus of future research.

## Conclusion

This review highlights the broad roles of Hh signaling in vascular biology. First, Hh signaling regulates multiple aspects of vasculogenesis; Ihh regulates vascular ECs induction with the mediation of Bmp4 [23]. Shh acts through the non-classical Hh pathway to alter ECs shape and accelerate migration [11, 85]. In addition, Hh signaling influences both physiological and pathological angiogenesis, but the effect can be positive or negative depending on including organ vascular development, ischemia, injury and tumor-related angiogenesis; however, the role of Hh signaling in different settings should be viewed as a dichotomy. Finally, the Hh pathway is associated with arterial remodeling by enhancing VSMCs differentiation and proliferation.

Therefore, Hh-related genes, antagonists, and agonists could be used to target different vascular diseases, provided that their potential side effects are better understood. This requires a fuller picture of the pathway’s underlying mechanisms, particularly the involvement of both canonical and non-canonical Hh signaling in regulating vascular biology. Further research will allow us to build consensus regarding the roles of these pathways and provide a firm empirical basis for developing treatments targeting vascular diseases.

## Data Availability

No datasets were generated or analysed during the current study.

## Abbreviations

Hh: Hedgehog
EC: Endothelial cell
VSMC: Vascular smooth muscle cell
Shh: Sonic hedgehog
Ihh: Indian hedgehog
Ptch: Ptched
Gli: Glioma-associated oncogene homolog
Smo: Smoothened
Sufu: Suppresser of fused
PKA: Protein kinase A
VEGF: Vascular endothelial growth factor
MSC: Mesenchymal stem cell
BMP: Bone morphogenetic protein
MMP: Matrix metalloproteinase
OPN: Osteopontin
Foxf: Forkhead transcription factor
Ang: Angiopoietin
FGF: Fibroblast growth factor
PVC: Perivascular cell
PTHrP: Parathyroid hormone-related protein
cNCC: cranial Neural Crest Cell
IRADE: Insulin resistance adipocyte-derived exosome
PDGF: Platelet derived growth factor
HGF: Hepatocyte growth factor
IGF: Insulin-like growth factor
SDF-1α: Stromal-derived-factor-1α
Klf2: Kruppel-like factor 2
TYMP: Thymidine phosphory lase
OSCC: Oral squamous cell carcinoma
CYR61: Cysteine-rich angiogenic inducer 61
CK: Chronic kidney disease
